# Supplementary material for: Structural basis for energy transduction by respiratory alternative complex III
Source: Nat Commun. 2018 Apr 30;9:1728. doi: 10.1038/s41467-018-04141-8 (PMC5928083; doi:10.1038/s41467-018-04141-8)
Supplement: Supplementary file 1 — Supplementary Information [file 41467_2018_4141_MOESM1_ESM.pdf]

Supplementary Information

Structural basis for energy transduction by respiratory alternative complex III

Sousa et al.

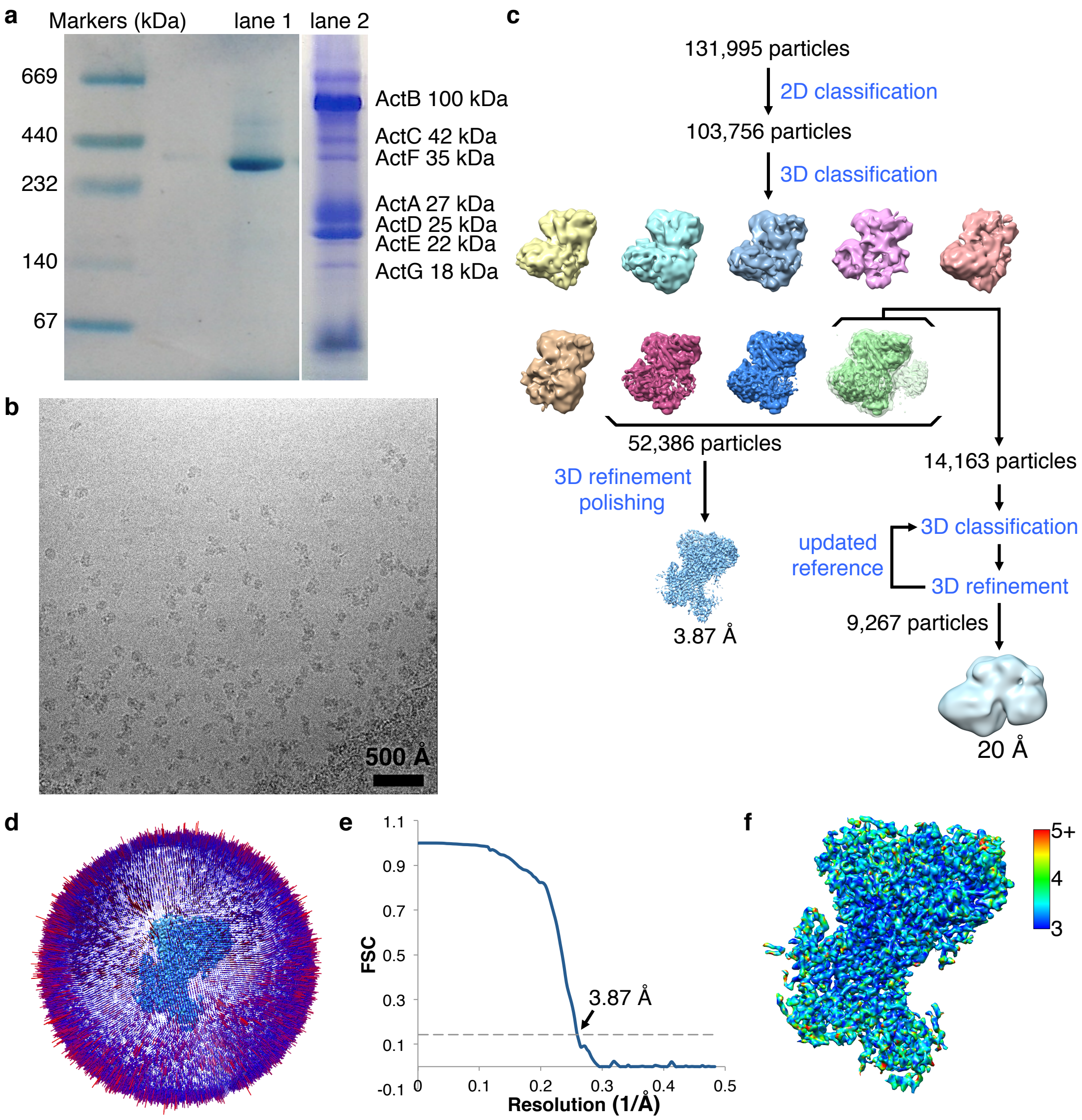

Supplementary Figure 1 | Cryo-EM data acquisition and image processing.
a, Blue Native-PAGE (lane 1) and SDS-PAGE (lane 2) of the ACIII sample used for cryo-EM stained with Coomassie. ACIII subunits are labeled, as identified previously[8,13]. The band at high MW results from an incomplete denaturation of the protein due to its stability. The lowest "band" in the gel corresponds to the sample front. ActH is expected to migrate with a predicted MW of ~20 kDa. No bands are observed for the *caa*$_3$ oxygen reductase subunits since this only accounts for less than 3% of the proteins in the sample. **b**, Representative electron micrograph of ACIII, collected with an energy-filtered Titan Krios (FEI/Thermo Fisher Scientific) at 300 kV on a Gatan K2 Summit electron detector. **c**, Approximately 132,000 particles were used for reference-free 2D classification in RELION. Particles from the best 2D classes were further

classified in 3D, using a low-resolution map generated in EMAN2 as initial reference. Independent 3D classifications consistently produced a class with a weak density at the membrane level (green 3D class), visible at low contour level (light green). Exhaustive 3D classification, after re-extraction of particles with a larger box size, yielded a small population with an additional density that was refined to 20 Å resolution. Almost 40 % of the initial particles fell within three high-quality 3D classes. Refinement of these particles produced a map with an overall resolution of 3.87 Å. **d**, 3D plot of angular distribution of the particles in the final ACIII map. **e**, Gold-standard FSC plot for the final refined map. Resolution estimated at FSC=0.143. **f**, ACIII map coloured according to local resolution determined by ResMap.

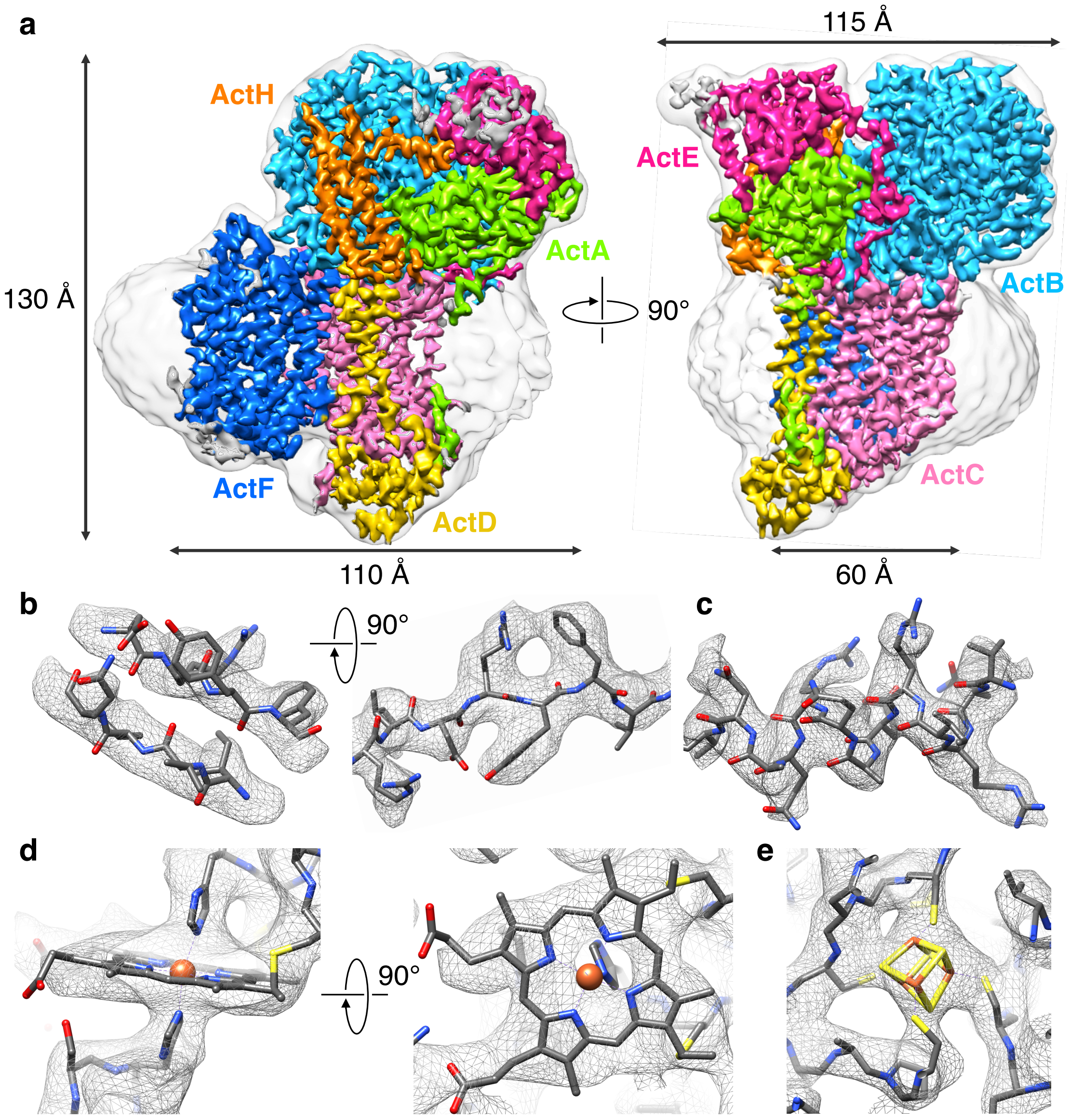

Supplementary Figure 2 | Near-atomic resolution cryo-EM map of ACIII. a, Side views of ACIII cryo-EM density map at 3.9 Å (subunits coloured as in Fig. 1) superimposed with a map low-pass filtered to 10 Å (transparent light grey) for visualization of the DDM micelle. The density of the eighth subunit of the complex, above ActE, is shown in grey. **b**, Cryo-EM density of a β-sheet, showing clear strand separation and good side chain densities. **c**, Cryo-EM density of an α-helix, with clear helical pitch and good side chain density. **d**, Cryo-EM density of heme II and its axial histidines. **e**, Cryo-EM density of cluster FeS2 and coordinating cysteines.

a

ActG (Rhom172_0210**):**

Sequence coverage: 52 %
Unique peptides: 20
Sequest score: 205.34

```
        10         20         30         40         50
MAEQEKKQPA VAEAELPAVQ PDEANFEAPR LYRMIWITVV LLAVISVGLV
        60         70         80         90        100
VLTQWQMQQN AEVAAATARY PLREETEAHA RQLLEGYGVV DAEQGVYRVP
       110        120        130
IDRAMEEIVE AYGGDSVWTL PQPSAVSRRM
```

b

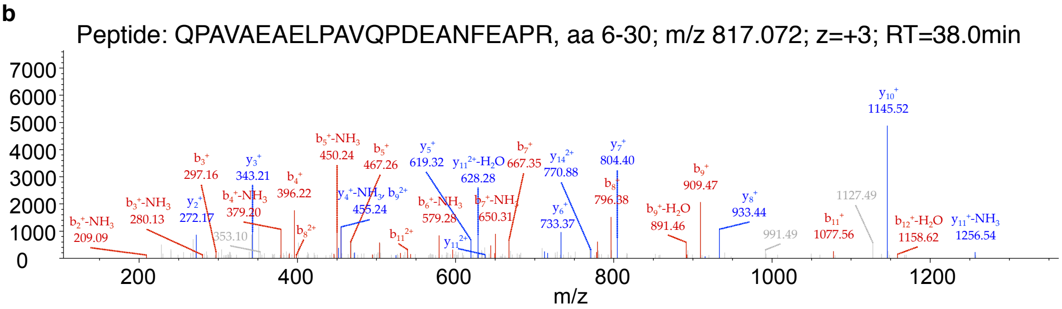

Supplementary Figure 3 | Identification of ActG by mass spectrometry. a, ActG (Rhom172_0210, Uniprot identifier: G2SEG6) identification using LC-MS/MS (sequence coverage: 52%, matched peptides highlighted in red). **b**, Annotated MS/MS spectrum for peptide QPAVAEAELPAVQPDEANFEAPR, corresponding to amino acid residues 6-30 of ActG.

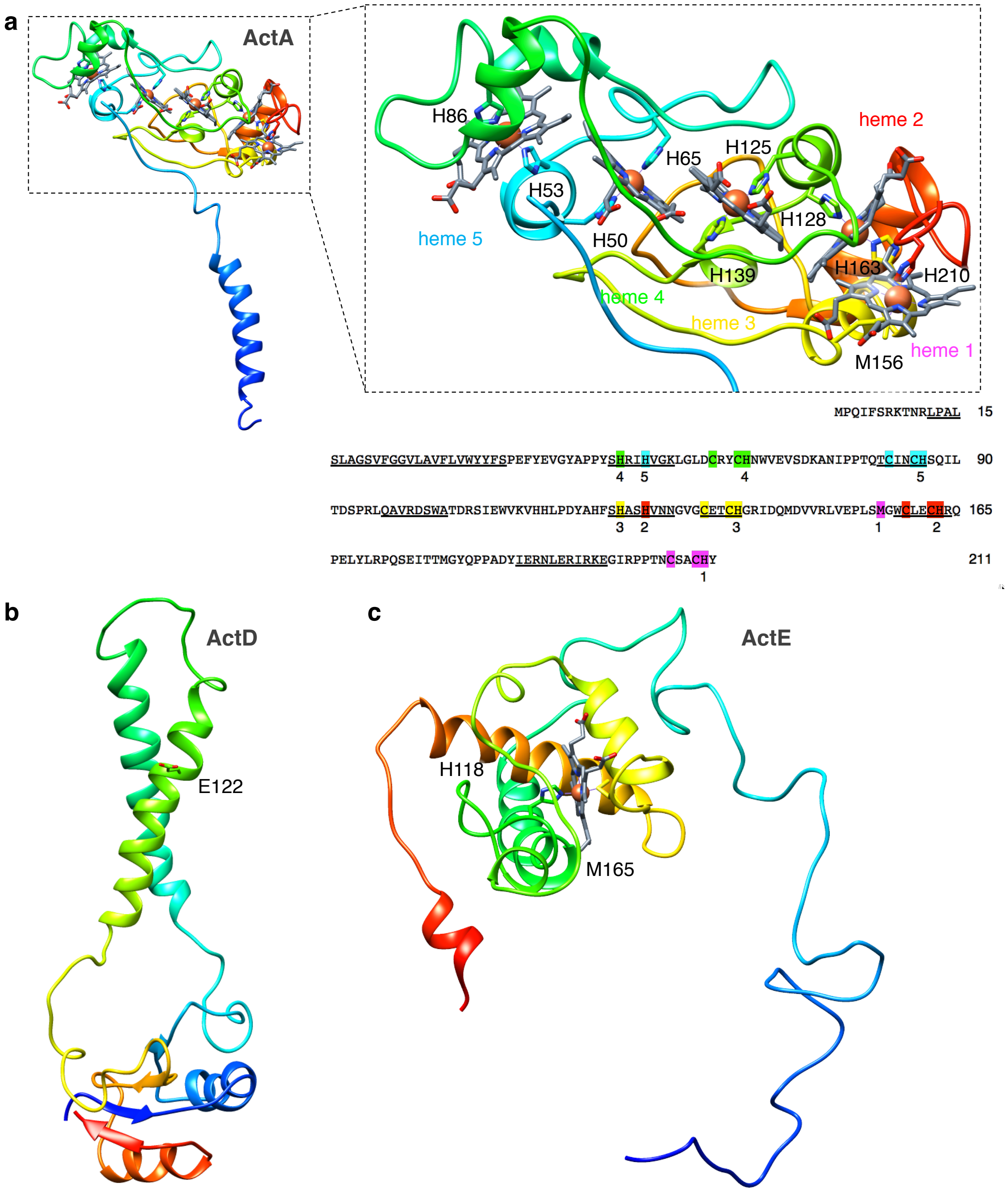

Supplementary Figure 4 | Structures of ACIII subunits. a, ActA is anchored to the membrane by one TMH. Its five hemes are each coordinated by a CxxCH motif and a distal residue (indicated in the sequence in a different color for each heme). The domains binding hemes 3-2 and 4-5 seem to be the result of gene duplication, reflected in a local two-fold pseudo-symmetry centered at hemes 3 and 4. **b**, ActD as seen from ActC. Its two TMH are arranged as a helix dimer, with the conserved Glu122 (see the text) in the centre of the membrane. Both termini have a cytoplasmic βαβ motif; the four strands form an antiparallel β-sheet. **c**,

The N-terminus of ActE forms a long loop that embraces ActB and reaches the membrane surface (Fig. 1). The heme in the C-terminal domain is coordinated by His118 and Met165. All structures are rainbow-coloured from blue (N-terminus) to red (C-terminus).

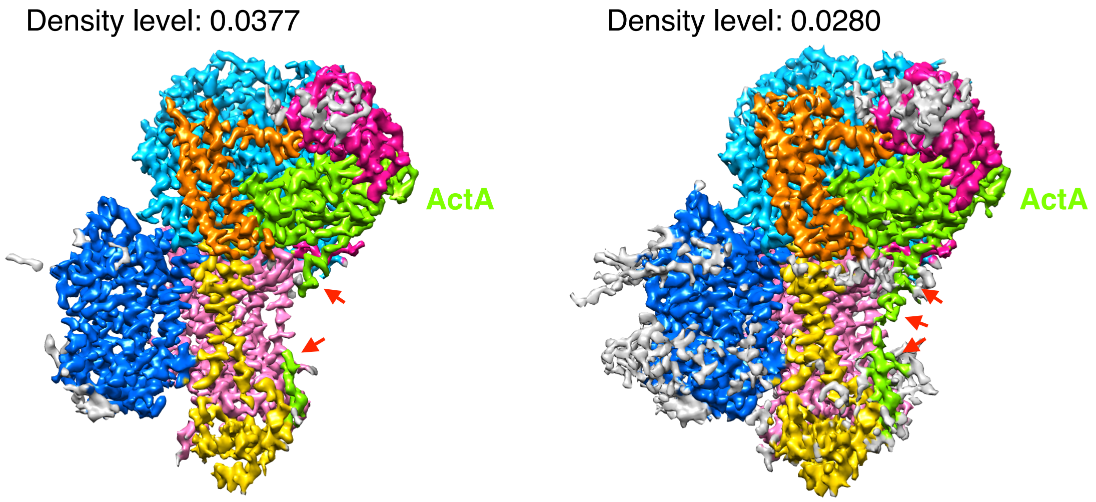

Supplementary Figure 5 | ActA TMH. ACIII map at two contour levels reveals a weaker density for TMH of ActA (indicated by red arrows). Subunits are colour-coded as in Fig. 1. Detergent was partially removed for clarity (grey density).

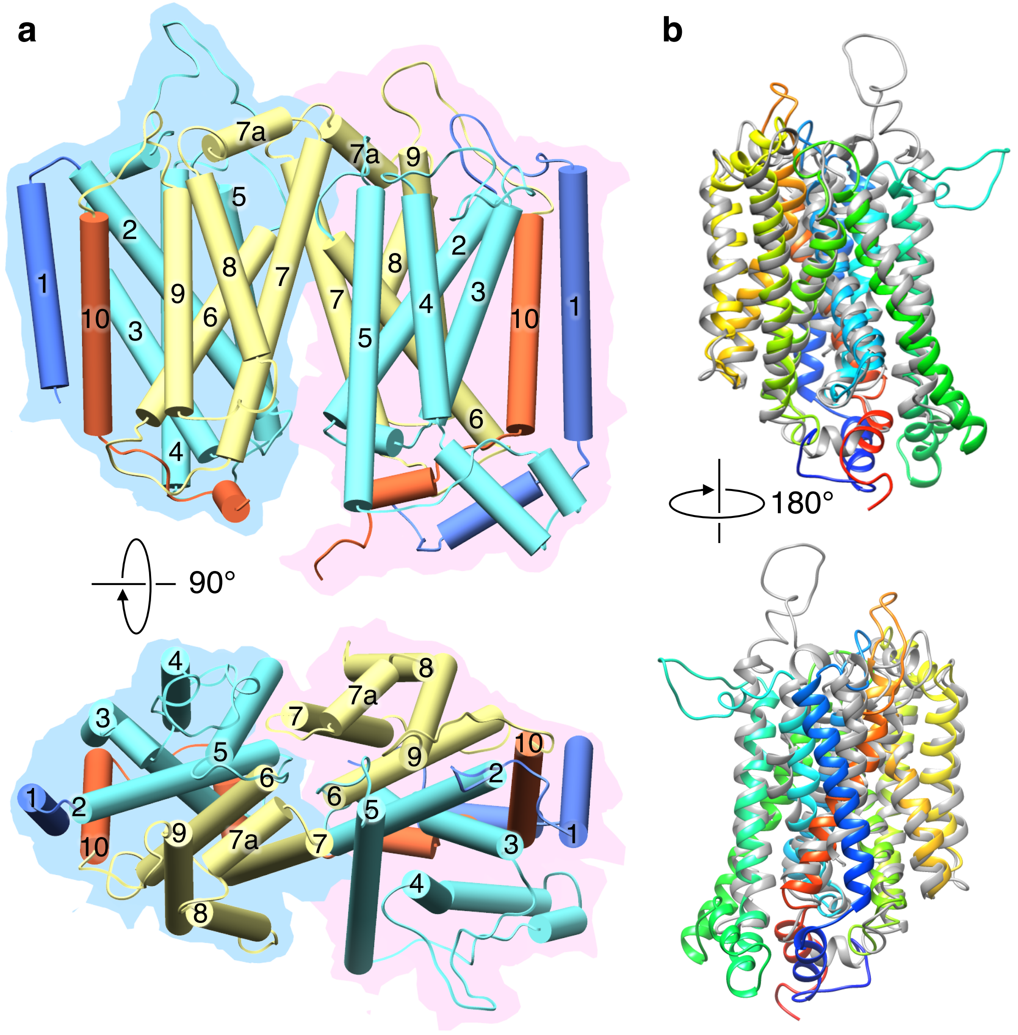

Supplementary Figure 6 | Fold of homologous membrane subunits ActC and ActF. a, ActC (pink silhouette) and ActF (light blue silhouette) seen from the membrane (top) and from the periplasm (bottom). Helices are represented as cylinders. The N- and C-terminal helices, which form a helix dimer, are coloured in blue and red, respectively. The four-helix bundles are shown in light blue (TMHs 2-5) and yellow (TMHs 6-9). **b**, Superposition of ActC and ActF indicates the same fold. ActC and ActF are represented as ribbons rainbow-coloured (blue, N-terminus; red, C-terminus) and in grey, respectively.

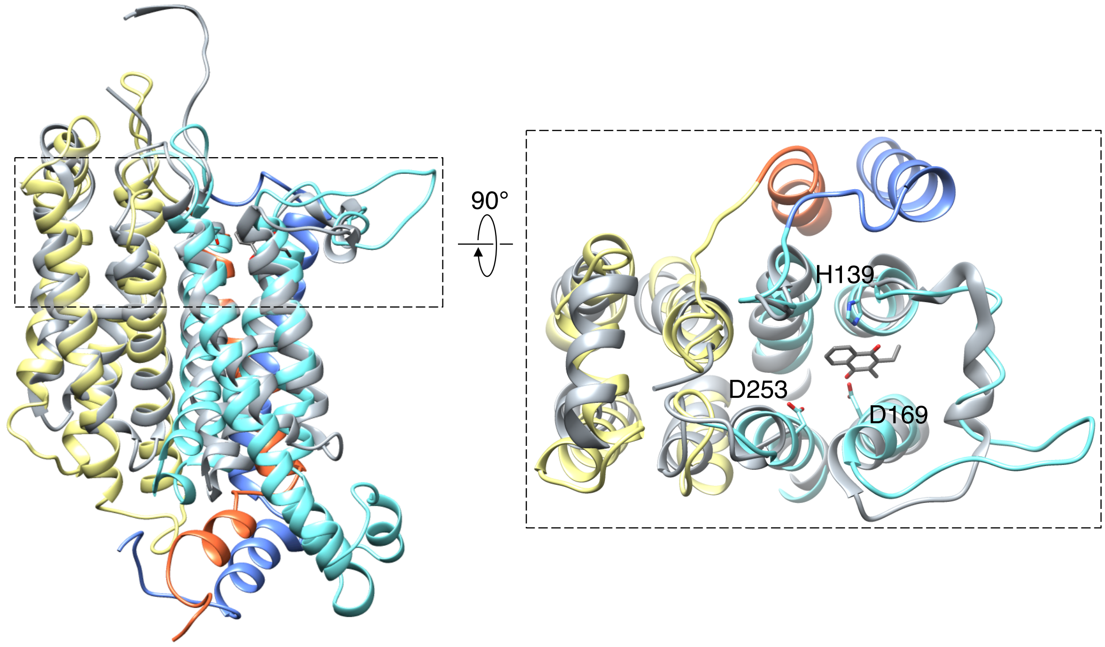

Supplementary Figure 7 | Structural homology of ActC and PsrC.

Superposition of ActC (coloured as in Supplementary Fig. 6a) and PsrC co-crystallized with menaquinone (PDB 2VPW; grey)[21] as seen from the membrane (left) and periplasm (right). Residues proposed to form part of the quinol-binding site in ActC are shown.

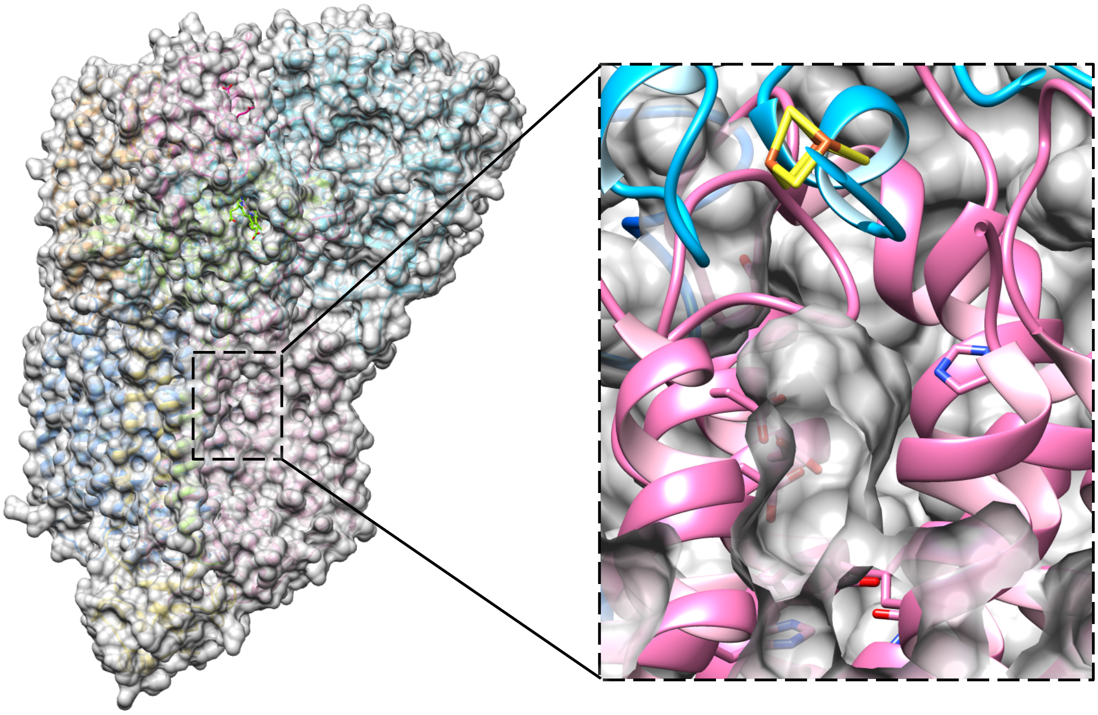

Supplementary Figure 8 | Entry channel to quinol-binding pocket. Putative quinol-binding site on the periplasmic side of ActC with surface representation (left) and zoomed view of the entry channel to the quinol-binding pocket (right). Subunits are coloured as in Fig. 1.

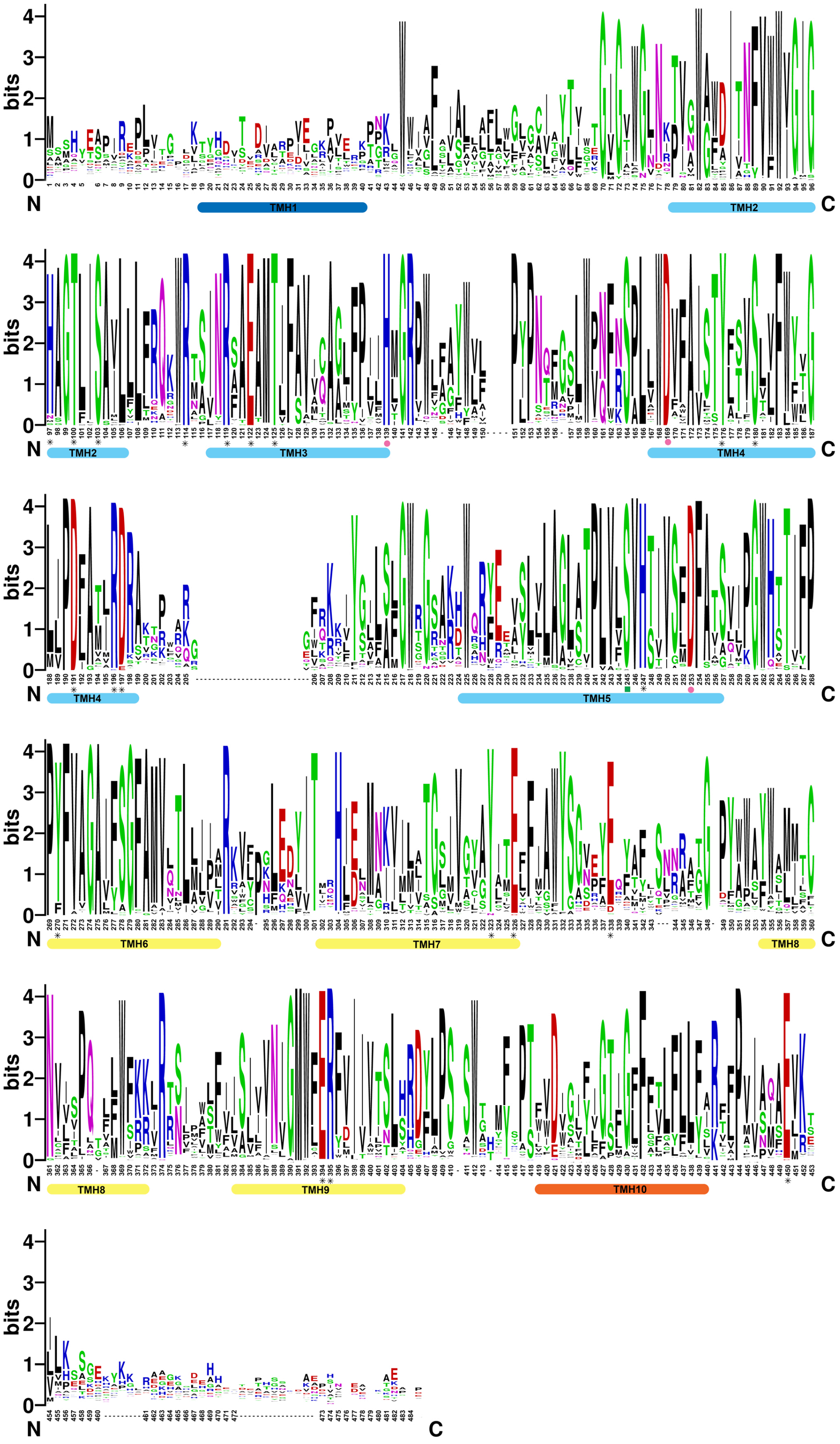

Supplementary Figure 9 | Sequence conservation in ActC. Sequences were retrieved as described previously[1,12]. Sequences from 146 different species were used in the alignment. Amino acid residues forming TMHs are indicated by

cylinders, using the same colour code as in Supplementary Fig. 6a. Residues from the putative proton half-channels and quinol-binding site are highlighted with asterisks (*) and pink dots, respectively. The residue involved in subunit cross-talk (*S*245) is indicated by a green square.

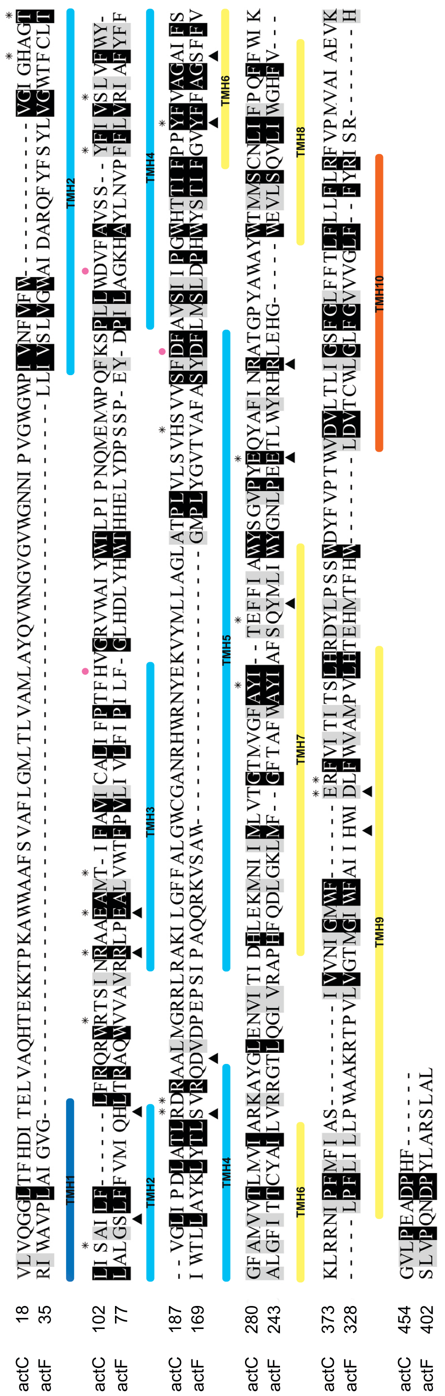

Supplementary Figure 10 | Structured-based sequence alignment of ActC and ActF. Amino acid residues forming transmembrane helices are indicated by cylinders, using the same colour code as in Supplementary Fig. 6a. Residues from the putative proton half-channels in ActC and ActF are marked by asterisks (*) and black triangles, respectively. Residues forming the quinol-binding site are indicated by pink dots.

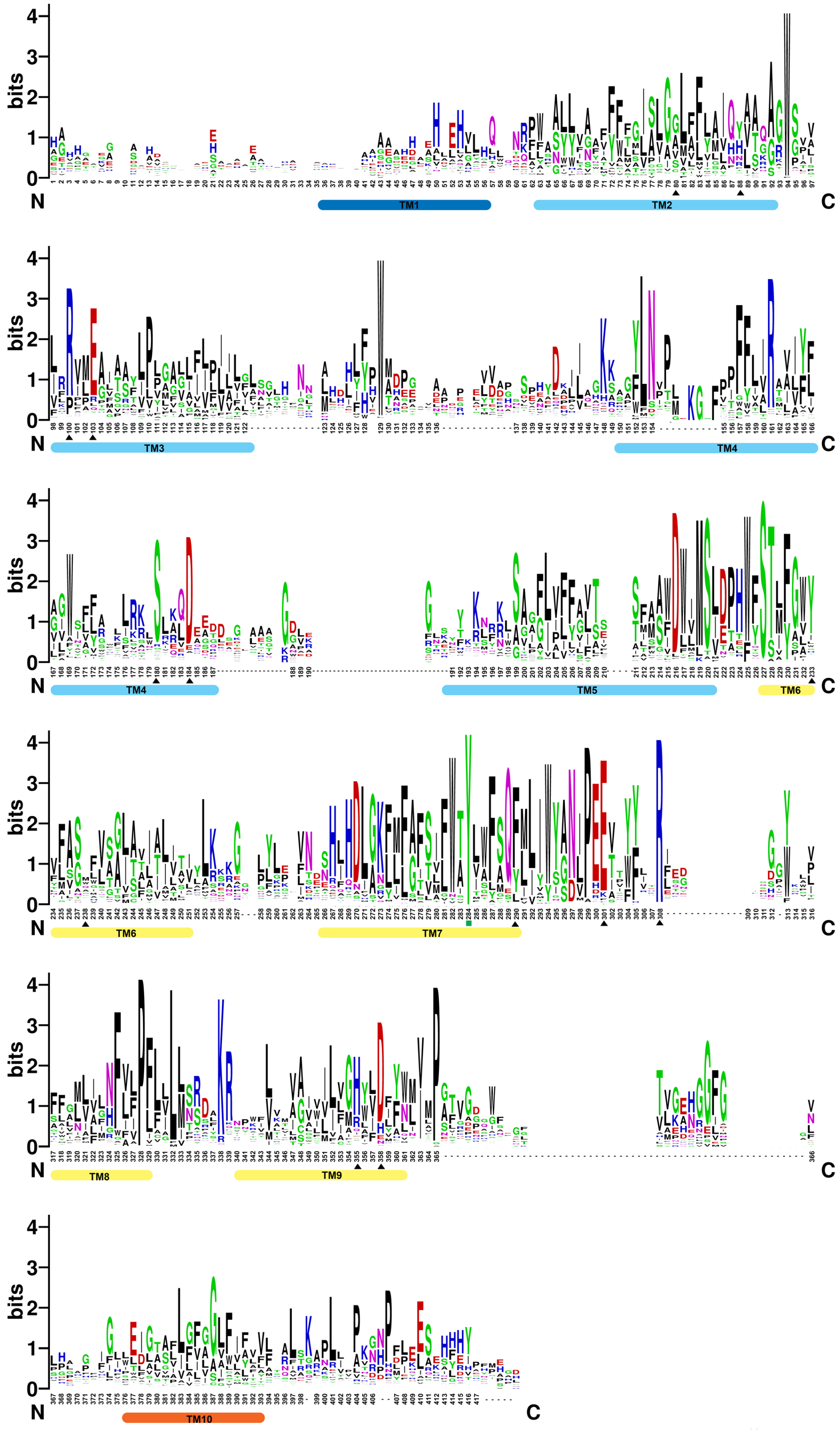

Supplementary Figure 11 | Sequence conservation in ActF. Sequences were retrieved as described previously[1,12]. Sequences from 136 different species were

used in the alignment. Amino acid residues forming TMHs are indicated by cylinders, using the same colour code as in Supplementary Fig. 6a. Residues forming the putative proton half-channels are marked by black triangles. The residue involved in subunit cross-talk (Y284) is indicated by a green square.

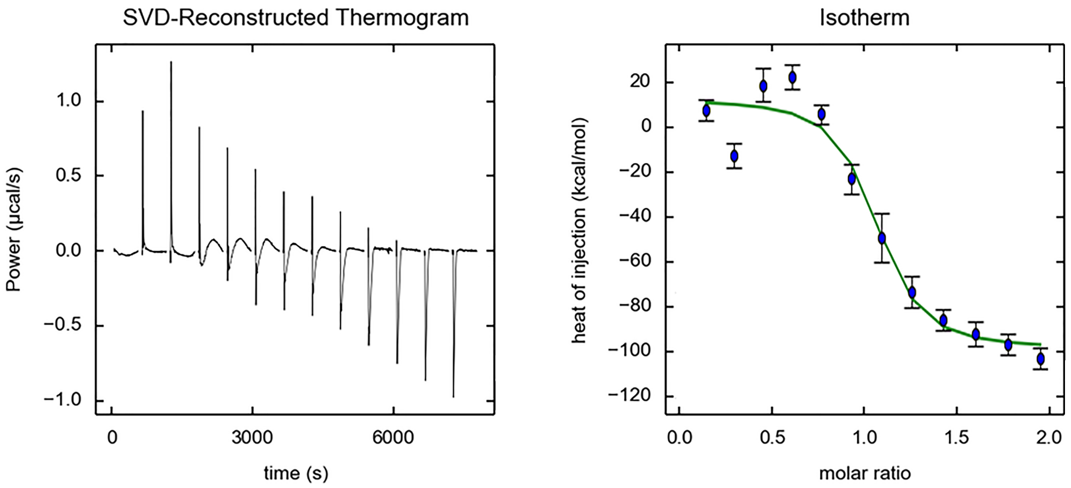

Supplementary Figure 12 | Quinone binding to ACIII detected by Isothermal Titration Calorimetry. Thermogram obtained by titrating 190 µM DMN to 19 µM ACIII at 65 °C, the optimal growth temperature of *R. marinus* (left) and data after processing using NITPIC and fitted with a 1:1 binding model, shown as a green line (right), with a binding constant (log K_a) of 6.5 ± 0.14.

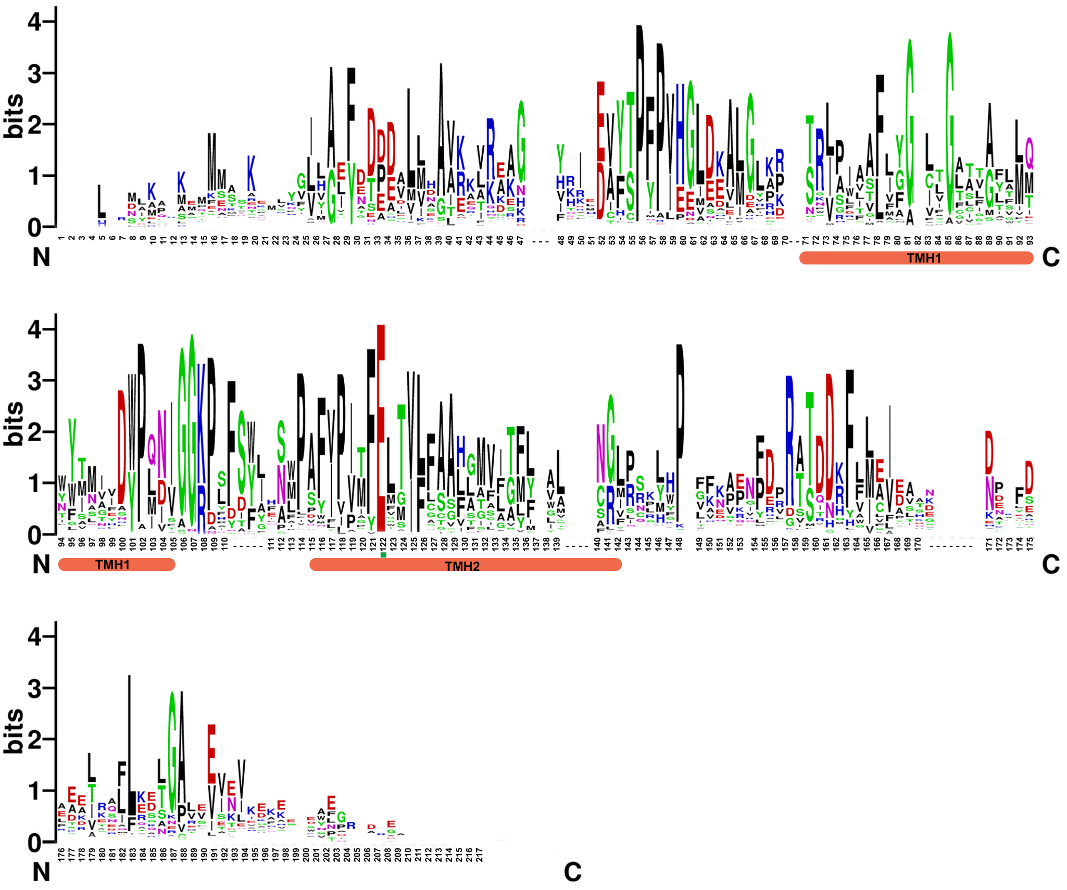

Supplementary Figure 13 | Sequence alignments of ActD. Sequences were retrieved as described previously[1,12]. Sequences from 138 different species were used in the alignment. Amino acid residues forming TMHs are indicated by orange cylinders. The residue involved in subunit cross-talk (E122) is indicated by a green square.

Supplementary Table 1 | Cryo-EM data collection and model statistics

**Data collection and 3D reconstruction**	
Electron microscope	FEI Titan Krios
Electron detector	Gatan K2 Summit
Voltage	300 kV
Defocus range	-0.6 to -4.0 µm
Pixel size	1.035 Å
Electron dose	72 $e^-/Å^2$
Initial particle images	131,995
Final particle images	52,386
Symmetry imposed	C1
Resolution	3.87 Å
B factor	-106 $Å^2$
**Model composition**	
Non-hydrogen atoms	20,287
Protein residues	2,503
Ligands	6 *c*-type hemes, 3 [4Fe-4S] clusters, 1 [3Fe-4S] cluster
***B* factors**	
Protein	75.04 $Å^2$
Ligand	53.98 $Å^2$
**R.m.s. deviations**	
Bond lengths	0.01 Å
Bond angles	1.06°
**Rotamers**	
Favoured	97.50 %
Allowed	2.5 %
Outliers	0 %
**Ramachandran plot**	
Favoured	86.26 %
Allowed	13.74 %
Outliers	0 %
**Validation**	
EMRinger score	1.37
MolProbity score	2.11 (100th percentile)
MolProbity clashscore, all atoms	8.85 (97th percentile)
