# Supplementary material for: Structural basis for energy transduction by respiratory alternative complex III
Source: Nat Commun. 2018 Apr 30;9:1728. doi: 10.1038/s41467-018-04141-8 (PMC5928083; doi:10.1038/s41467-018-04141-8)
Supplement: Supplementary file 3 — Description of Additional Information [file 41467_2018_4141_MOESM3_ESM.docx]

**Description of Additional Supplementary Files**

File Name: Supplementary Movie 1

Description:

ActH fit in electron density map. Cryo-EM density map of ActH with fitted model rainbow-coloured from blue (N-terminus) to red (C-terminus).

File Name: Supplementary Movie 2

Description:

Quinol pocket and proton pathways in ACIII. Key residues identified in the ACIII structure are displayed sequentially. The quinol-binding site and respective entry channel are shown using surface representation, seen from the membrane and from the periplasm. Residues facing the interior of the corresponding four-helix bundle and proposed to form two half-channels in ActC and ActF are shown while slicing through the structure in a plane parallel to the membrane. Back at the central plane of the membrane, where the cytoplasmic and periplasmic half-channels meet, the conserved residues Glu122^D^, Ser245^C^ and Tyr284^F^ are positioned near Asp169^C^ part of the quinol-binding site. Finally, the relative position of the residues listed above is observed from the membrane. Density for the side chains of Asp169^C^, Asp253^C^ and Glu122^D^ is not visible; these have been modelled. A yellow sphere indicates the location of the entry point to the quinol pocket. Subunits are coloured as in Fig. 1.
